# Spatial Congruity Effects Reveal Metaphorical Thinking, not Polarity Correspondence

**DOI:** 10.3389/fpsyg.2015.01836

**Published:** 2015-11-26

**Authors:** Sarah Dolscheid, Daniel Casasanto

**Affiliations:** ^1^Department of Rehabilitation and Special Education, University of Cologne, Cologne, Germany; ^2^Department of Psychology, The University of Chicago, Chicago, IL, USA

**Keywords:** conceptual metaphor, markedness, musical pitch, polarity correspondence, space

## Abstract

Spatial congruity effects have often been interpreted as evidence for metaphorical thinking, but an alternative account based on polarity correspondence (a.k.a. markedness) has challenged this view. Here we compared metaphor- and polarity-correspondence-based explanations for spatial congruity effects, using musical pitch as a testbed. In one experiment, English speakers classified high- and low-frequency pitches as “high” and “low,” or as “front” and “back,” to determine whether space-pitch congruity effects could be elicited by *any* marked spatial continuum. Although both pairs of terms describe bipolar spatial continuums, we found congruity effects only for high/low judgments, indicating that markedness is not sufficient to produce space-pitch congruity effects. A second experiment confirmed that there were no space-pitch congruity effects for another pair of terms that have clear markedness (big/small), but which do not denote spatial height. By contrast, this experiment showed congruity effects for words that cued an appropriate vertical spatial schema (tall/short), even though these words are not used conventionally in English to describe pitches, ruling out explanations for the observed pattern of results based on verbal polysemy. Together, results suggest that space-pitch congruity effects reveal metaphorical uses of spatial schemas, not polarity correspondence effects.

## Introduction

Are *high* hopes somewhere in the air? Or what about *rising* prices? And where exactly are *low* numbers? Spatial metaphors like these are very common in language. According to theories of metaphorical mental representation (e.g., Lakoff and Johnson, 1980), people not only talk metaphorically but also *think* metaphorically, activating mental representations of space to scaffold their thinking in a variety of non-spatial domains. Whereas initial arguments in favor of metaphor theory were based on linguistic data, numerous psychological experiments have now shown that spatial representations contribute to people’s understanding of non-spatial domains like time (Torralbo et al., 2006), social dominance (Schubert, 2005), emotional valence (Meier and Robinson, 2004), similarity (Casasanto, 2008), and musical pitch (Rusconi et al., 2006; for reviews, see Landau et al., 2010; Casasanto and Bottini, 2014a).

Many of these psychological studies base their findings on binary stimulus-stimulus or stimulus-response compatibility tasks. In one experiment, for instance, participants were asked to classify dimensions in a metaphoric target domain (e.g., valence: judge the positive or negative valence of a word), while, at the same time aspects of the spatial source domain were varied (i.e., location of the stimuli: top or bottom of the screen). Consistent with “GOOD is UP” metaphors in language, people were faster to evaluate positive words when they appeared in a high spatial location compared to a low location (and vice versa for negatively-valenced words; Meier and Robinson, 2004). Similarly, participants made faster judgments about social power when words for powerful people were at the top of a display and powerless people at the bottom (e.g., “king” above “slave,” Schubert, 2005), and not vice versa. These metaphor congruity effects, with faster performance for congruent compared to incongruent trials, have been taken as evidence that metaphoric target domains automatically activate congruent spatial information, supporting Lakoff and Johnson’s (1980) conceptual metaphor theory.

On an alternative account, however, it has been argued that spatial congruity effects may be better explained in terms of *polarity alignment* (Proctor and Cho, 2006), also called *markedness* (Clark, 1969; Lakens, 2012; Lynott and Coventry, 2013). Like many other continuums in language and mind, metaphoric “source domains” like *height* and “target domains” like *happiness* tend to be marked, or bipolar. That is, they consist of an unmarked or +polar endpoint (e.g., *high*, *happy*), and an opposing marked or –polar endpoint (*low*, *sad*). Unmarked endpoints (+polar) are commonly defined as the default, and are generally broader or more evaluatively positive than marked (–polar) endpoints (Lehrer, 1985; Proctor and Cho, 2006; for a critique see Haspelmath, 2006). The constructs of “polarity” and “markedness” are generally discussed by different communities of researchers, but these constructs are overlapping and may be impossible to distinguish, even terminologically; like polarity theorists (Proctor and Cho, 2006), markedness theorists typically describe unmarked poles as “positive” and marked poles as “negative” (Clark, 1973). Here we will use the terms “markedness” and “polarity” interchangeably.

The markedness of continuums in language and mind can often be traced to asymmetries in the body and world. Clark (1973) explains how the specifics of human perceptual organs, and of our habitat, give rise to the markedness of two spatial continuums that serve as source domains for whole families of metaphors: the up/down continuum and the front/back continuum. The unmarked (+polar) poles of these continuums are those that we can perceive more easily, whereas the marked (–polar) poles are harder to perceive. Clark (1973) notes that “since everything above ground level is perceptible and nothing below it is, upward is naturally positive and downward is naturally negative” (p. 33). Likewise, “the front of an object is the facet that contains the perceptual apparatus, as in animals, whereas the back is that facet which does not. The front is the direction toward which an animal moves, and the back is the direction from which an animal moves; front is the positive direction, and back is the negative direction” (ibid, p. 43). Clark argues that the markedness/polarity of these spatial continuums gets extended to non-spatial domains that people talk about using spatial metaphors in language, and by hypothesis *think* about using schematic spatial representations (e.g., domains like time, valence, and pitch).

Whether or not a perceptual basis for markedness is apparent, the marked and unmarked ends of continuums can be identified on the basis of patterns in language. The unmarked (+polar) term often serves as the name of the entire continuum: people’s physical stature is measured on a continuum of *height*, not of *shortness*. Accordingly, the unmarked term can describe values along the whole continuum: a flagpole can be “10 meters high,” but not “10 meters low” (see also Clark et al., 1973). Questions posed using unmarked term make no presupposition, whereas questions using the marked term presuppose an answer in a restricted range of the continuum. For example, “How *tall* is he?” does not presuppose that a person is tall. The answer could be “He is average height” or even “He is a midget.” By contrast, “How *short* is he?” presupposes an answer on the short end of the continuum. Markedness also constrains the order in which the two poles of a continuum tend to be mentioned in verbal collocations. Turning to the front–back continuum, Landsberg (1995) notes that “the front [is] the most important, and hence we will always think—and speak—of “front first,” using sequences like *in front of-behind*, *before-after*, *to-from*, *front–back*, and shunning such serials as *behind-in front of, *after-before, *from-to, *back-front, etc.” (p. 70, italics added).

There is abundant evidence that markedness/polarity alignment can affect cognitive processing. Participants show faster reaction times (RTs) for unmarked (+polar) dimensions compared to marked (–polar) ones, and faster RTs when poles are aligned than when they are misaligned (see Clark, 1969; Seymour, 1974; Proctor and Cho, 2006). In light of the evidence for markedness effects, it is important to consider whether RT benefits for congruent metaphoric source-target pairings (like *happy* and *up*) could be explained by an additive processing advantage for +polar endpoints (e.g., happy +polar, up +polar)—without any need to invoke metaphor theory. Does polarity alignment offer an alternative, non-metaphorical explanation for metaphor congruity effects like those reported by Meier and Robinson (2004), Schubert (2005), and many other studies that rely on dimensional compatibility in binary speeded response tasks (Lakens, 2012)? And if so, what would this mean for theories of metaphorical mental representation?

Crucially, not all of the evidence for metaphoric thinking comes from binary response-time congruity effects. A variety of methods have been used, and studies show that people’s metaphoric representations of domains like time and musical pitch map onto space in a continuous analog fashion (Casasanto and Boroditsky, 2008; Dolscheid et al., 2013). English speakers, for instance, who talk about musical pitch in terms of spatial height (e.g., “high” vs. “low”) also implicitly associate higher pitches with higher positions in space in non-linguistic psychophysical tasks. In one study, participants were asked to reproduce musical pitches while watching lines that varied in spatial height. Lines of nine different heights were fully crossed with nine different pitches. Participants’ pitch reproductions were affected by this irrelevant spatial information throughout the ranges of pitches and heights presented. On average, the same tones were reproduced at the lowest frequencies when they were accompanied by the lowest lines, at the highest frequencies when they were accompanied by a highest lines, and at intermediate frequencies when they were accompanied by lines of intermediate spatial heights; these results show a continuous linear relationship between spatial height and pitch [Dolscheid et al., 2013; see Casasanto and Boroditsky (2008) for an analogous result showing a continuous spatial mapping of temporal duration]. In this study, responses were not speeded, the dependent variable was not RT, and the metaphor-congruity effects did not rely on the kind of binary stimulus-response compatibility that is believed to give rise to polarity alignment effects (Proctor and Cho, 2006).

Further impetus to question whether markedness can account for metaphor congruity effects comes from the fact that some mappings between space and musical pitch appear to go *against* markedness. Whereas speakers of many languages (including English) refer to pitch in terms of spatial height, other languages use different metaphors (e.g., Eitan and Timmers, 2010). Speakers of Farsi or Turkish, for instance, describe pitch in terms of spatial thickness (Shayan et al., 2011). These thickness-pitch metaphors show reversed polarity alignment compared to height-pitch metaphors. Thick (+polar) refers to a low frequency pitch (–polar), whereas thin (–polar) refers to a high frequency pitch (+polar). Farsi speakers implicitly represent pitch in terms of thickness, consistent with their verbal metaphors (Dolscheid et al., 2013); in this case, where markedness and metaphorical spatial schemas are in opposition, spatial schemas predicted people’s behavior, and markedness/polarity alignment did not.

Although experiments like Dolscheid et al.’s (2013) provide evidence for metaphorical mental representation that cannot be explained by markedness, the role of markedness in binary compatibility tasks remains controversial. Do source-target congruity effects merely show polarity alignment? Or do they reveal metaphoric associations? Although metaphors and polarity are often indistinguishable in spatial compatibility tasks (see Lakens, 2012), we predict that when markedness and metaphor are juxtaposed, spatial congruity effects will support metaphoric thinking, not markedness. What should primarily determine the pattern of responses is whether the stimuli that participants have to classify in binary compatibility tasks activate a metaphor-appropriate spatial schema (e.g., in the case of space-pitch mappings for English speakers, it should be a uni-dimensional vertical spatial schema). That is, we propose that schema-appropriateness should be necessary, and markedness should not be sufficient, to produce congruity effects between metaphorical source and target domains.

In Experiment 1, we tested compatibility between space and pitch for two pairs of spatial terms, both of which describe paradigm cases of marked spatial continuums (Clark, 1973). One pair (high/low) corresponds to the poles of the correct vertical spatial continuum, which English speakers use to think about pitch, and the other pair (front/back) to the poles of an incorrect spatial continuum, which is not used for pitch in English (or any other known language). As explained above, “high” and “front” both constitute the unmarked or +polar endpoint, whereas “low” and “back” represent the marked or –polar endpoint of spatial continuums (Clark, 1973; Landsberg, 1995). The markedness of the pitch continuum has been questioned by some researchers, and it is possible that its markedness varies across contexts (Eitan and Timmers, 2010; Eitan, 2013). But if markedness is to explain binary height-pitch compatibility effects that are predicted by height-pitch metaphors in English, which have been reported previously (e.g., Melara and Marks, 1990; Rusconi et al., 2006), then the high-frequency pole of the pitch continuum must be processed as unmarked (+polar), and the low-frequency pole as marked (–polar)—at least in the context of spatial height. Here participants were asked to make binary speeded judgments on high-frequency and low-frequency pitches, classifying pitches either in a polarity-congruent way (e.g., high pitches as “high” or “front”), or in a polarity-incongruent way (e.g., high pitches as “low” or “back”). If polarity alignment drives space-pitch congruity effects, then similar effects should be found when pitch is mapped to any marked linear spatial continuum, regardless of its orientation: High/low and front/back should both produce pitch-congruity effects. Alternatively, if activating a particular spatial schema for pitch is critical (i.e., the schema that is encoded in verbal metaphors the participants’ language), then high/low should result in a congruity effect, but front/back should not.

## Experiment 1

### Materials and Methods

#### Participants

Twenty-four English speakers with no reported hearing problems participated for payment (5$ per 30 min). Four participants were excluded from analyses for not following instructions (i.e., they responded according to the wrong response mapping throughout at least one task). They were replaced by a new sample of 4 participants who had not previously participated in the task. This study was carried out in accordance with the recommendations of New School for Social Research (NSSR) Institutional Review Board (IRB) with written informed consent from all subjects. All subjects gave written informed consent in accordance with the Declaration of Helsinki.

#### Materials and Procedure

Participants performed two tasks: categorizing pitches as high vs. low (high/low task) or as front vs. back (front/back task). In both tasks participants heard tones, one at a time, and were asked to categorize each tone as quickly and accurately as possible by pressing buttons on the QWERTY keyboard (Q and P keys). Stimuli were presented on an Apple iMac using Vision Egg 2.6 (Straw, 2008). Tones were generated by Audacity software^1^ and comprised two pure tones (low frequency tone: 262; high frequency tone: 440 hertz). Each tone lasted 400 ms. Tones were presented at approximately 60 dB-a. At this amplitude, standard equal loudness curves suggest the perceived loudness of our high and low pitches should be nearly identical. Differences in subjective loudness levels between the two tones, therefore, are unlikely to account for any observed congruity effects.

Participants listened to tones via sealed headphones (Sennheiser HD201). Immediately following the offset of each tone, two response options (e.g., high, low) appeared, one on the bottom left and the other on the bottom right of the screen. Participants were instructed to classify the sound by pressing the button located under the corresponding word (e.g., high or low) as fast and accurately as possible. The left–right locations of the spatial terms varied randomly from trial to trial so that participants could not predict the location of the correct word in advance, and so that there was no association between left–right space and pitch or spatial height.

Tasks (high/low and front/back) were presented in 2 blocks. Within each block, spatial terms were crossed with pitches to create 2 different space-pitch pairings (e.g., Congruent: categorizing high-frequency pitches as “high”; Incongruent: categorizing high-frequency pitches as “low”). The order of task blocks was counterbalanced across participants. The congruent and incongruent trials were also presented in blocks, with the order of congruity blocks counterbalanced within each task block. Across task blocks, incongruent and congruent conditions were always presented in alternation. Before each condition, participants received 6 practice trials with feedback. Participants were also given an example illustrating the appropriate space-pitch pairing before the practice trials.

Each condition consisted of 24 trials, yielding 48 trials per task and 96 trials in total for each participant. For half of the trials a high pitch was presented, for the other half a low pitch was presented, in each condition. The order of high and low pitches was randomized. In the high–low congruent condition, the high pitch had to be categorized as *high* and the low pitch as *low*. In the high–low incongruent condition, the high pitch had to be categorized as *low* and the low pitch as *high*. In the front–back congruent condition the high pitch had to be categorized as *front* and the low pitch as *back* (according to patterns of polarity/markedness). In the front–back incongruent condition the low pitch had to be categorized as *front* and the high pitch as *back*. Participants’ responses immediately initiated the next trial.

#### Results

All data were analyzed using R (version 2.14.2)^2^ and the R packages *lme4* (Bates and Maechler, 2009) and *languageR* (Baayen, 2007; cf. Baayen, 2008). We carried out linear mixed effects regression models of Space (high–low versus front–back) and Congruity (congruent, incongruent) on accuracy and RTs. Using the principle of backward selection, we started out with a full (conservative) model which took into consideration not only the random intercept but also the random slopes of subject whenever it was appropriate (i.e., when the factor was a within-subject factor). Random intercepts and slopes of items were not included in the analysis due to the small number of items (4 words: high/low, front/back). To interpret the significance, we adopted the criterion that a given cosine was significant if the absolute value of the *t*-statistic (or *z*-statistic) exceeded 2 (Baayen, 2008).

***Accuracy***

The mean accuracy for all target trials was 92.4% (SD = 8.1). For the high/low task, accuracy was 92.4% (SD = 9.7), and for the front/back task, accuracy was 92.5% (SD = 11.3). For congruent conditions, accuracy was 95.9% (SD = 4.5) and for incongruent conditions it was 88.9% (SD = 15.5). Analyzing accuracy by using a logistic mixed effects model on binary accuracy data yielded no main effects or interaction of Space (high/low, front/back) and Congruity (congruent, incongruent), (Space: *z* = |1.3|; Congruity: *z* = |0.2|; Space by Congruity: *z* = |1.2|).

***Reaction times***

Reaction times of the button presses were analyzed by linear mixed effects models. Only correct trials were analyzed which resulted in the exclusion of 7% of the data. Responses greater or less than ± 2 SDs away from each participant’s average RTs were also excluded, which resulted in the removal of 6% of the accurate trials.

**FIGURE 1:**
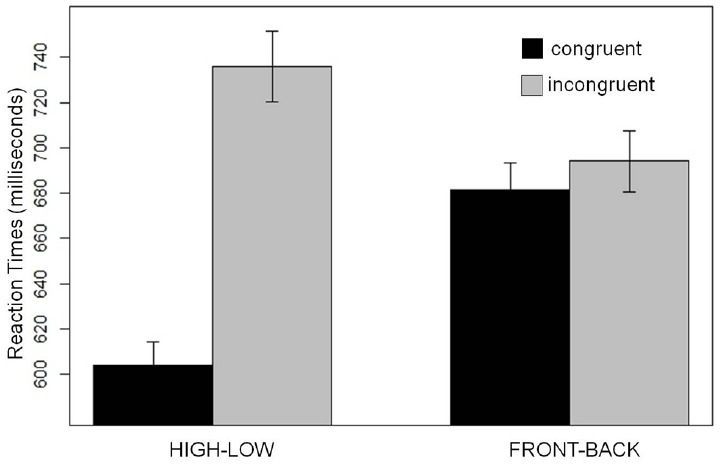
**Experiment 1 results.** The influence of Space (high–low; front–back) and Congruity (congruent; incongruent) on pitch categorization (plotted in milliseconds). A significant congruity effect was found in the high–low task (left) but not in the front–back task (right). Error bars indicate the SEM.

Of primary interest, there was significant interaction of Congruity by Space (*t* = |3.3|). This interaction comprised a significant effect of Congruity in the high/low task (*t* = |4.5|), but not in the front–back task (*t* = |0.2|; Figure 1). There was also a significant main effect of Congruity (*t* = |3.5|), but this effect was not of interest for discriminating between the “metaphor” and “markedness/polarity-correspondence” hypotheses.

### Discussion

In Experiment 1 we find congruity effects for the high/low but not for the front/back task, suggesting that activating the appropriate spatial schema (i.e., spatial height) is what is relevant in such binary response compatibility tasks. Words that activate a different (irrelevant) spatial schema (front–back), however, do not result in a congruity effect. This finding indicates that congruity effects cannot be attributed to markedness (polarity alignment), since “front” and “back” also name the unmarked and marked ends of a (sagittally oriented) linear spatial continuum.

## Experiment 2

Whereas high/low terminology is conventional for pitch in English, front/back is not. Maybe we only found a congruity effect for high/low in Experiment 1 because this space-pitch mapping is lexicalized, whereas front/back is not? This skeptical interpretation of the results would not change the fact that markedness was not sufficient to elicit congruity effects, but it would call into question our claim about activating the right spatial schema. Do we find congruity effects only because participants were using the polysemous words high/low, which can refer to “height” in both space and pitch?

To rule out this explanation, in Experiment 2 we compared congruity effects for two pairs of spatial terms: tall/short and big/small. Neither pair of spatial expressions can be used in conventional English to describe the height of musical pitches (i.e., their frequency). If high/low congruity effects were driven by polysemy, then neither of these pairs of spatial terms should produce a congruity effect. However, if space-pitch congruity effects result from using words that activate a vertical spatial schema, then “tall” and “short” should produce a congruity effect because they are *schematically appropriate* (even though they are *lexically inappropriate*). By contrast, “big” and “small” should not produce any space-pitch congruity effect, because these terms refer to 3-dimensional size, and should not activate the appropriate 1-dimensional vertical spatial schema (Dirven and Taylor, 1988; Taylor, 2002).

In addition to testing whether the height-pitch congruity effect in Experiment 1 depended on the polysemy of “high” and “low,” Experiment 2 also provides a second test of the sufficiency of markedness to produce space-pitch congruity effects. “Big” is the unmarked (+polar) end and “small” the marked (–polar) end of the big-small spatial continuum. Therefore, assuming that the high-frequency and low-frequency poles of the pitch continuum are the unmarked poles, respectively, then markedness/polarity correspondence makes a clear prediction: judgments should be faster when “big” is matched with “high” than when “small” is matched with “high.” This is not the only possible prediction that can from markedness, however. Eitan (2013) and Eitan and Timmers (2010) have proposed that the markedness of pitch may be context dependent: perhaps, in the context of big vs. small space, the markedness of the high- and low-frequency poles reverse. Whether or not one agrees with this proposal, it is important to note that it makes an alternative prediction: on a “reversed-markedness” account, judgments in our big/small task should be faster when “small” is matched with “high” than when “big” is matched with “high.” Both of these markedness-based predictions contrast with the prediction that follows from the vertical space-pitch metaphors in English: we should find a significant congruity effect for the word pair that activates a uni-dimensional vertical spatial schema (tall/short), but no significant effect (or a significantly weaker effect) for the word pair that activates a different spatial schema (big/small).

### Materials and Methods

#### Participants

Twenty-four English speakers who did not participate in Experiment 1, and who had no reported hearing problems participated for payment (5$ per 30 min). One participant was excluded from analyses for not following instructions (i.e., the participant responded according to the wrong response mapping throughout one condition). He was replaced by a new participant. This study was carried out in accordance with the recommendations of NSSR IRB with written informed consent from all subjects. All subjects gave written informed consent in accordance with the Declaration of Helsinki.

#### Materials and Procedure

The same procedure as in Experiment 1 was used, with the following exceptions. Rather than classifying pitches as high–low or front–back, participants classified them as tall-short for one block and big-small for the other.

For the tall-short task, the high pitch had to be categorized as *tall* and the low pitch as *short* in the congruent condition, whereas the low pitch had to be categorized as *tall* and the high pitch as *short* in the incongruent condition. For the big-small task, we coded markedness according to the standard assumption that the high-frequency pole of the pitch continuum is unmarked: thus, in the condition we labeled as “congruent” the high pitch had to be categorized as *big* and the low pitch as *small*, whereas in the condition we labeled as “incongruent” the low pitch had to be categorized as *big* and the high pitch as *small*. Importantly, the decision to code congruity in this way did not affect our ability to detect space-pitch compatibility effects. According to this coding, an RT advantage for pairing high pitches with “big” would be consistent with standard assumption that high-frequency pole is unmarked end of the pitch continuum; alternatively, an RT advantage for pairing high pitches with “small” would be consistent with Eitan and colleagues’ suggestion that the markedness of pitch reverses in the context of spatial size. Either size-pitch congruity effect, therefore, would be consistent with a markedness-based prediction, and at odds with the prediction that follows from vertical spatial metaphors in English.

### Results

We carried out linear mixed effects regression models of Space (tall-short versus big-small) and Congruity (congruent, incongruent) on accuracy and RTs, as in Experiment 1. Using the principle of backward selection, we again started out with a full (conservative) model which took into consideration not only the random intercept but also the random slopes of subject whenever it was appropriate (i.e., when the factor was a within-subject factor). Random intercepts and slopes of items were not included in the analysis due to the small number of items (4 words: tall/short, big/small). To interpret the significance, we adopted the criterion that a given cosine was significant if the absolute value of the *t*-statistic (or *z-*statistic) exceeded 2 (Baayen, 2008).

#### Accuracy

The mean accuracy for all target trials was 94.8% (SD = 11.4). For the tall/short task, accuracy was 94.6% (SD = 13.1), and for the big/small task, accuracy was 94.9% (SD = 10.1). For congruent conditions, accuracy was 96.2% (SD = 6.7) and for incongruent conditions it was 93.3% (SD = 17.1). Analyzing accuracy by using a logistic mixed effects model on binary accuracy data yielded no main effects or interaction of Space (tall/short, big/small) and Congruity (congruent, incongruent), (Space: *z* = |1.0|; Congruency: *z* = |1.0|; Space by Congruity: *z* = |1.0|).

#### Reaction Times

Reaction times of the button presses were analyzed by linear mixed effects models. Only correct trials were considered which resulted in the exclusion of 4% of the data. Responses greater or less than ± 2 SDs away from each participant’s average RTs were also excluded, which resulted in the removal of 4% of the accurate trials.

**FIGURE 2:**
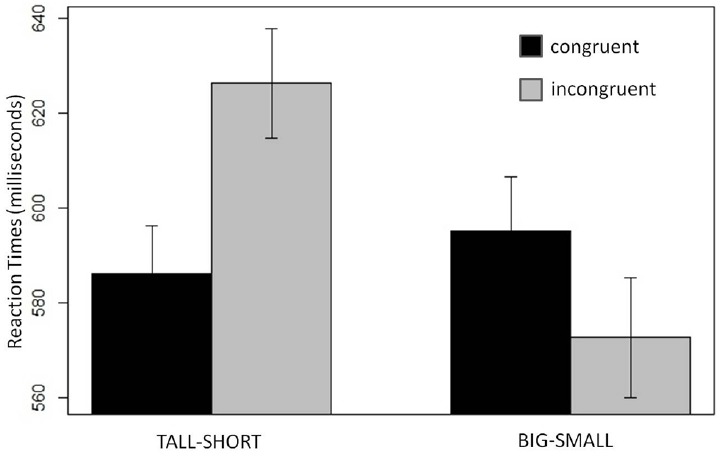
**Experiment 2 results.** The influence of Space (tall–short; big–small) and Congruity (congruent; incongruent) on pitch categorization (plotted in milliseconds). A significant congruity effect was found in the tall–short task (left) but not in the big-small task (right). Error bars indicate the SEM.

Of primary interest, there was a significant interaction of Congruity by Space (*t* = |3.2|). This interaction comprised a significant effect of Congruity in the tall/short task (*t* = |3.0|), but not in the big/small task (*t* = |1.5|; Figure 2).

### Discussion

In Experiment 2 we found a congruity effect in the tall/short task but not in big/small task. Therefore, the vertical space-pitch congruity effects found in Experiments 1 and 2 cannot be attributed to polysemy (since “tall” and “short” do not denote pitch in English), nor to markedness (since there were no effects of alignment between the poles of the high/low frequency continuum and the big/small spatial continuum). Rather, space-pitch congruity effects resulted from activating the appropriate spatial schema, which serves as a metaphorical scaffold for English speakers’ mental representations of musical pitch.

Results of the big/small task in Experiment 2 both corroborated and complemented those of the front/back task in Experiment 1. Front/back is a marked linear spatial continuum, like high/low, but these continuums are anchored by different minimum values or “zero points”; whereas the zero point for high/low is at the negative (marked) pole, the zero point for front/back is mid-way between the negative (marked) and positive (unmarked) poles. We have no reason to believe that pitch cannot be mapped to a spatial continuum that is anchored in the middle (in fact, there is evidence that some musicians spatialize pitch on a left–right continuum; Rusconi et al., 2006). In principle, however, the difference in 0 points between the spatial continuums tested in Experiment 1 could account for the observed pattern of positive and null results. Yet, this skeptical possibility is ruled out by the results of Experiment 2; like the high/low continuum, the big/small continuum is anchored by a 0 point at the negative pole.

Two aspects of the design of Experiment 2 bear careful consideration. First, as discussed in Section Materials and Methods, not all researchers agree on which pole of the pitch continuum is marked in the context of spatial size (Eitan and Timmers, 2010; Eitan, 2013). Does this ambiguity create a methodological problem for the present experiment? No. According to our experimental hypothesis verticality, not markedness, is the “active ingredient” in the dominant space-pitch mapping in English speakers’ minds. According to the alternative hypothesis, markedness is the active ingredient in mappings between space and non-spatial domains. The possibility that either the high-frequency *or* the low-frequency end of the pitch continuum is marked in the context of spatial size meant that, in our big/small task, there were *two* possible congruity effects that could be interpreted as support for a markedness-based account—stacking the deck in favor of the markedness hypothesis. Yet, even so, neither of these patterns was found. As in Experiment 1, a space-pitch congruity effect was only found where it was predicted by vertical space-pitch metaphors in English.

Second, we note that although we found no significant size-pitch congruity effects here, relationships between size and pitch have been reported previously (e.g., Evans and Treisman, 2010). It is clear that, at least under some circumstances, English speakers may implicitly associate larger sizes with lower pitches. However, on the basis of our previous research, we would predict that all else being equal, the height-pitch mapping should be stronger or more automatically activated in English speakers’ minds than the size-pitch mapping (if the latter is found, at all).

As an alternative to height-pitch metaphors, some languages have conventional size-pitch metaphors, and other languages have thickness-pitch metaphors (Eitan and Timmers, 2010; Shayan et al., 2011). Thickness is one measure of multidimensional spatial size. A thickness-pitch mapping is, therefore, similar to a size-pitch mapping; in the limit, size-pitch and thickness-pitch expressions in language could correspond to the same 3-dimensional spatial schema scaffolding pitch representations. We have found previously that 4-month old infants are sensitive both to the height-pitch mapping that is encoded in linguistic metaphors in English and to the thickness-pitch mapping that is encoded in other languages like Farsi (Dolscheid et al., 2014). By adulthood, however, our experiments show that only one of these mappings is evident in people’s non-linguistic pitch judgments. Dutch speakers’ pitch estimates were influenced significantly by irrelevant spatial height information, but not by irrelevant size/thickness information. Conversely, Farsi speakers’ pitch estimates were influenced significantly by irrelevant size/thickness information, but not by irrelevant spatial height (Dolscheid et al., 2013).

How could infants who are sensitive to both height-pitch and size/thickness-pitch mappings turn into adults who preferentially activate only one of these mappings when they represent pitch? This process can be understood in terms of *hierarchical mental metaphors theory* (HMMT; Casasanto and Bottini, 2014b). According to this proposal, the implicit, non-linguistic source domain-target domain mappings (a.k.a. mental metaphors) that people tend to use most often are specific members of a more general family of source domain-target domain mappings. Development of mental metaphors appears to occur over a two-stage process. First, in the case of space and pitch, a superordinate “family” of mappings is established that includes the height-pitch and size/thickness-pitch mappings (it remains an open question whether size-pitch and thickness-pitch mappings are best considered to be “siblings” or one and the same). These mappings may be constructed on the basis of observable correlations between space and pitch in the natural world, over ontogenetic or phylogenetic time. The height-pitch mapping reflects the fact that people involuntarily raise their larynxes, chins, and sometimes other body parts (e.g., eyebrows) when they produce higher pitches, and lower them when they produce lower pitches (Miller, 1986). The size/thickness mapping reflects a pervasive correlation between pitches and the size of the objects or creatures that produce them: consider the different ranges of pitches produced by thin vs. thick strings on a guitar; big vs. small drums; large vs. small animals; etc. Data from infants suggests not only that height-pitch and size/thickness-pitch mappings are both present pre-linguistically, but also that the mappings are about equally strong; Dolscheid et al. (2014) found no difference in the strength of the looking-time congruity effects for height-pitch vs. size/thickness-pitch stimuli.

When children learn metaphors in language, a second process begins. Our findings in adults suggest that each time people use a linguistic metaphor like “a high pitch” they activate the corresponding mental metaphor, strengthening this mapping at the expense of competing mappings in the same “family” of space-pitch associations. As a consequence, speakers of “height languages” like Dutch and English come to rely on vertical spatial schemas to scaffold their pitch representations more strongly than multidimensional spatial schemas, whereas speakers of “thickness languages” like Farsi come to rely on multidimensional spatial schemas, more strongly than vertical spatial schemas (Dolscheid et al., 2013). According to HMMT, the process of strengthening certain mental metaphors via the use of the corresponding linguistic metaphors results in the *weakening* of other members of the family of mappings—but does not cause these dispreferred mappings to be extinguished. Consistent with this suggestion, Dolscheid et al. (2013) showed that Dutch speakers could be induced to use a non-linguistic thickness-pitch mapping (like Farsi speakers) after only a brief training intervention.

Together, Dolscheid et al. (2013) experiments in infants and adults suggest that speakers of a “height language” like English may possess a mental metaphor linking pitch with size/thickness, but that this mapping should be substantially weaker than the mental metaphor linking pitch with height, which gets strengthened through the use of conventional height-pitch metaphors in English. It should be unsurprising, then, that size-pitch mappings should be found in some task contexts but not in others (see Dolscheid et al., 2013), and that in the present experiment we found a significant height-pitch congruity effect but no significant size-pitch congruity effect, despite the fact that the height-pitch and size-pitch tasks were otherwise matched and equipotent.

Importantly, for the purposes of the present study, the possibility of a weak or latent metaphorical mapping between size and pitch in English speakers’ minds worked *against* our prediction that congruity effects should be found when pitch was crossed with vertical space but not with sagittal space or 3-dimensional size.

## Experiment 3

For Experiment 3 we did not collect any new behavioral data. Instead, we submitted the results of the high/low and tall/short task from Experiments 1 and 2 to further analysis, adapting a method Lakens (2012) proposed to discriminate between metaphor theory and polarity correspondence. Although these analyses use some of the data from Experiments 1 and 2, they take a different approach to testing our main claim: that the space-pitch congruity effects are driven by metaphorical mental representation, not by markedness/polarity alignment. Our approach in Experiments 1 and 2 was to contrast pitch congruity effects in tasks involving uni-dimensional vertical spatial continuums (i.e., high/low and tall/short) with the lack of congruity effects in tasks involving other marked spatial continuums (i.e., front/back and big/small). Our strategy in Experiment 3 is to probe the details of the vertical space-pitch congruity effects we found to determine whether they are uniquely consistent with metaphor theory, or whether they are consistent with markedness/polarity alignment, as well.

In many cases, metaphor theory and markedness/polarity correspondence make overlapping predictions. In the present experiments, metaphor theory and polarity correspondence made contrasting predictions for the front/back and big/small tasks, but both theories predicted that high pitches should be congruent with high/tall, and low pitches with low/short. In the analyses we have presented so far, the results of the high/low and tall/short tasks appear equally consistent with metaphor and with markedness/polarity alignment. Yet, further analyses of these results could constrain how these results bear on the two theories that we seek to disentangle.

**FIGURE 3:**
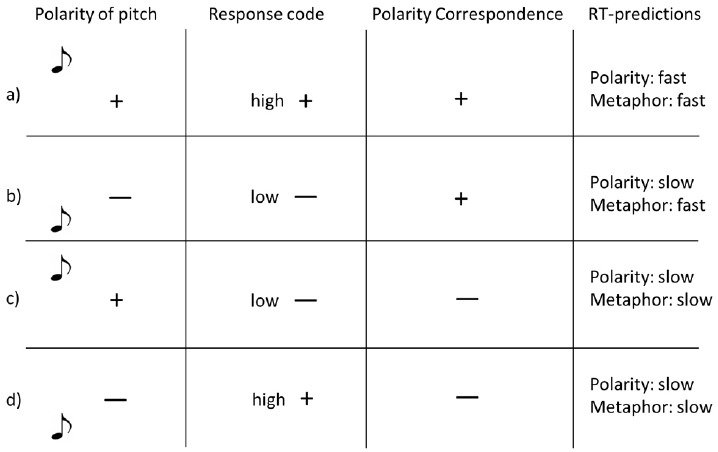
**Response time predictions of polarity correspondence vs. metaphor accounts, according to **Lakens (2012)****.

Lakens (2012) reasoned that, in speeded binary compatibility tasks, both metaphor theory and polarity correspondence predict a statistical interaction between polar continuums like vertical space and pitch, but that the details of the interaction can discriminate between the theories. Specifically, he reasoned that although metaphor and polarity correspondence make similar RT predictions for three of the four cells in a 2 × 2 design (e.g., Space: high, low; Pitch: high, low; see Figure 3 for an overview), they make different predictions in a fourth cell, which is therefore of critical importance (Figure 3,b). Theories of markedness/polarity predict that, relative to the other conditions, RTs should be slow when participants classify stimuli at the –polar ends of both continuums (i.e., low pitch, low space): despite the fact that the poles are aligned in this condition, negative polarity stimuli are predicted to be processed more slowly than positive polarity stimuli (Clark, 1969). By contrast, Lakens reasoned that according to metaphor theory responses in this cell should be relatively fast since low pitch and low space are metaphorically congruent. On the basis of this reasoning, previous studies have interpreted results that show no congruity effects in the negative polarity conditions to be evidence for polarity correspondence and against metaphor theory (Lakens, 2012; Lynott and Coventry, 2013).

Yet, this reasoning is faulty where it concerns metaphor theory, as Lakens has subsequently acknowledged (Santiago and Lakens, 2015). On tasks like we report here, the only prediction that can be derived from metaphor theory is that there should be a 2-way interaction between the two levels of the source domain (e.g., height) and of the target domain (e.g., pitch). The details of this interaction cannot be predicted *a priori*—at least not on the basis of metaphor theory—and are likely to vary according to idiosyncrasies of the stimuli, task, participants, etc. The same critical interaction could be found with or without any significant main effects (e.g., with or without a main effect of spatial height or of pitch height), and with varying collections of significant or non-significant simple effects in the four pairwise comparisons between cells. In our high/low and tall/short tasks, *any pattern of main effects and simple effects* would support metaphor theory so long as the 2 × 2 interaction was significant and in the predicted direction (i.e., overall, metaphor-congruent responses must be faster than metaphor-incongruent responses). In short, the analysis method that Lakens (2012) proposed cannot be used to disconfirm the predictions of metaphor theory.

We agree, however, that Lakens (2012) analysis method *can* be used to disconfirm predictions of polarity correspondence/markedness. We conducted two sets of analyses in order to determine whether the high/low and tall/short congruity effects reported in Experiment 1 and 2 were compatible with a markedness/polarity correspondence account. Whereas metaphor theory makes no predictions about the details of these congruity effects, polarity correspondence predicts that congruity effects should be weakest in the –polar conditions (Lakens, 2012; Lynott and Coventry, 2013). Therefore, in the analyses below, if congruity effects are significantly weaker in the –polar conditions than in the +polar conditions, this outcome would be consistent with both metaphor theory and with markedness/polarity correspondence. By contrast, if congruity effects do not differ between the –polar conditions and +polar conditions, this outcome would still be consistent with metaphor theory, but would disconfirm the predictions of markedness/polarity correspondence.

### Methods and Results

Significant metaphor-congruity effects were already reported in Experiments 1 and 2, for both the high/low and tall/short tasks, respectively. Here we examined the details of these effects, testing whether the space-pitch congruity effects were significant for the low-pitch trials (–polar) and the high-pitch trials (+polar), considered separately, and critically, whether the magnitudes of the congruity effects differed between low- and high-pitch trials.

#### High/Low Task (Experiment 1)

**FIGURE 4:**
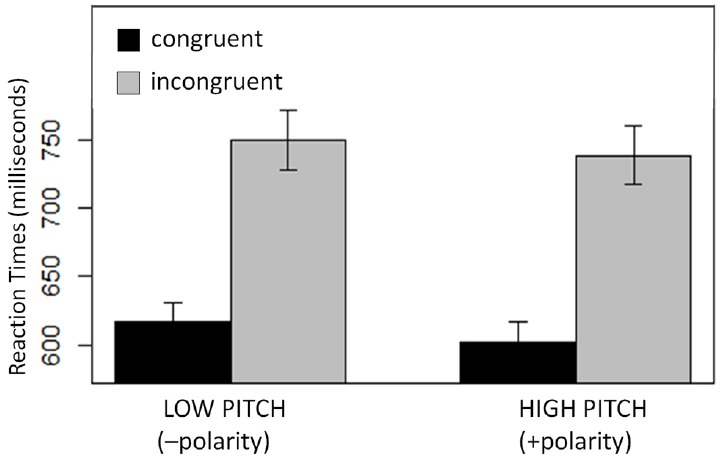
**Response time patterns of high–low congruity effects.** The influence of Congruity (congruent; incongruent) on pitch categorization for high pitches vs. low pitches (plotted in milliseconds). Error bars indicate the SEM.

First, a linear mixed effects regression of Congruity on RTs restricted to high pitches was conducted. Results showed a significant effect of Congruity (*t* = |4.7|; Figure 4, right). Next, the regression model was restricted to low pitches, and this model also revealed a significant effect of Congruity (*t* = |4.0|). That is, participants were faster to classify low pitches (–polar) as low (–polar) than to classify low pitches as high (Figure 4, left). The magnitude of these congruity effects did not differ significantly (*t* = |0.1|); that it, the observed space-pitch congruity effect was no stronger for high-pitch (+polar) trials than for the low-pitch (–polar) trials. This result is compatible with metaphor theory, but incompatible with the predictions of polarity alignment.

#### Tall/Short Task (Experiment 2)

**FIGURE 5:**
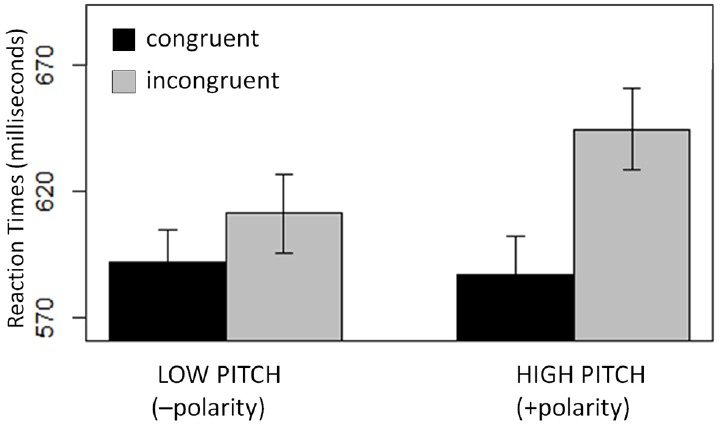
**Response time patterns of tall-short congruity effects.** The influence of Congruity (congruent; incongruent) on pitch categorization for high pitches vs. low pitches (plotted in milliseconds). Error bars indicate the SEM.

Analogous analyses were conducted for tall-short congruity effects of Experiment 2. Results showed a significant effect of Congruity (*t* = |3.0|; Figure 5, right) when high pitches had to be classified (+polar trials) but there was only a trend in the predicted direction when low pitches had to be classified (–polar trials; *t* = |1.2|; Figure 5, left). Critically, however, the magnitude of the effect in low pitch trials did not differ significantly from the effect found in high pitch trials (*t* = |1.4|). Again, this result is compatible with metaphor theory, but incompatible with the predictions of polarity alignment.

### Discussion

Significant metaphor-congruity effects were reported in Experiments 1 and 2. Metaphor theory licenses no predictions about the details of these effects, but polarity correspondence makes a clear prediction: congruity effects should be weakest in the –polar conditions (Lakens, 2012; Lynott and Coventry, 2013). Yet, this prediction was not upheld. In Experiment 1 the space-pitch congruity effect was highly significant for the low-pitch trials, and the magnitude of this effect did not differ from magnitude of the effect found in high-pitch trials. In Experiment 2 the space-pitch congruity effect only trended in the predicted direction for the low-pitch trials, but the magnitude of this effect did not differ significantly from the significant effect found in high pitch trials.

In principle, metaphor-congruity effects and polarity-alignment effects could co-occur, and their effects could combine. This combination would result in a strengthening of the metaphor congruity effects for the +polar trials, and a weakening of the metaphor congruity effects for –polar trials. Yet, no such pattern was found; there was no significant difference in strength between the +polar and –polar trials, in either the high/low or tall/short tasks.

Together, these analyses suggest that the high/low and tall/short congruity effects we found in Experiments 1 and 2 are not equally compatible with metaphor theory and polarity correspondence, even though both theories predicted the observed 2-way interactions of space and pitch. Upon examining the details of these interactions, we see that the results both confirm the predictions of metaphor theory and disconfirm the predictions of markedness/polarity correspondence. Since this was also the case for the results of the front/back and big/small tasks, we can conclude that overall, all four tasks of Experiments 1 and 2 are compatible with metaphor theory and incompatible with markedness/polarity correspondence.

## General Discussion

In three experiments, we examined relationships between space and musical pitch in binary response-time congruity tasks. We tested whether space-pitch congruity effects were best explained in terms of metaphor theory (Lakoff and Johnson, 1980) or polarity correspondence (a.k.a. markedness; Clark, 1969; Proctor and Cho, 2006). Classifying pitches with vertical spatial words (high vs. low; tall vs. short) elicited space-pitch congruity effects, but no comparable effects were found when participants were asked to classify pitches with words that name the poles of other marked spatial continuums (front vs. back; big vs. small). Polarity correspondence, alone, did not produce space-pitch congruity effects. Congruity effects emerged only when participants activated the vertical spatial schema encoded in space-pitch metaphors in English.

In many cases, the polarities of metaphorical source and target domains are aligned (Lakens, 2012). The unmarked ends of target domains like happiness, power, and pitch are all metaphorically UP (i.e., the unmarked end of this spatial continuum), whereas the marked ends of these continuums are metaphorically DOWN (i.e., the marked end of the spatial continuum). Therefore, in principle, congruity effects like those we found in our high/low and tall/short tasks could be equally consistent with metaphor theory and with markedness/polarity correspondence. Yet, further analyses showed that these congruity effects were *only* consistent with predictions of metaphor theory; they were inconsistent with the predictions of polarity correspondence.

Polarity correspondence predicted that the high/low and tall/short congruity effects should be weakest in the –polar conditions, but this was not the case. There was no significant difference in the strength of the congruity effects found in the –polar vs. +polar conditions in either the high/low or tall/short tasks, and the congruity effect in the –polar condition was highly significant in the high/low task. Together, these results show that markedness/polarity correspondence is neither necessary nor sufficient to produce the kind of binary response compatibility effects we found here, which were predicted on the basis of vertical space-pitch metaphors in English.

In our experiments the spatial source domain was not manipulated physically but rather via linguistic stimuli (see Dolscheid et al., 2013, for corroborating results from a non-linguistic task). Importantly, congruity effects were not restricted to polysemous words like “high” and “low,” which can be used for both space and pitch. Rather, congruity effects were also found for the words “tall” and “short,” which have no musical senses. Finding a significant space-pitch congruity effect in the tall/short task argues against a skeptical interpretation of the non-significant effects in the front/back and big/small tasks: it was not necessary for the stimulus words to be used conventionally to talk about pitch in English—the congruity effects did not depend on overlap in conventional words for space and pitch. Rather, it was both necessary and sufficient for participants to activate a vertical spatial schema.

Could congruity effects in tall/short tasks still be driven by polysemy *indirectly*? Could participants in the tall/short task have activated the words “high” and “low” covertly when classifying pitches, via semantic priming? This explanation is unlikely to account for our results, for several reasons. According to Latent Semantic Analysis (LSA)^3^, “tall” is more strongly related to “short” (LSA cosine: 0.48) than to “high” (LSA cosine: 0.31), and “short” is about equally strongly related to “high” (LSA cosine: 0.30) as to “low” (LSA cosine: 0.31). Since activation is expected to spread between the most strongly related items (Collins and Loftus, 1975), simple spreading activation would have wiped out a tall-short congruity effect rather than producing it.

Further evidence against an explanation of our findings based on semantic priming comes from the fact that “big” is more closely related to “high” than to “low” (LSA cosine: 0.18 versus 0.12), yet there was no congruity effect in the big/small task. The non-significant big/small effect trends in the *opposite direction* from this pattern of semantic associations (see Figure 2). To summarize these points, the LSA semantic distances between tall/short and high/low are not aligned in a way that should produce the predicted congruity effect, and yet a significant effect was found. By contrast, the semantic distances between big/small and high/low *are* aligned in a way that could produce a congruity effect via semantic priming from the stimulus words to the conventional space-pitch terms in English, and yet no such effect was found. This pattern argues strongly against an explanation of our tall/short congruity effect based on semantic priming of the words “high” and “low.”

Our results converge with those of a study showing space-time and space-number congruity effects that could not be explained by polarity correspondence (Santiago and Lakens, 2015). Markedness/polarity correspondence effects are well established (Clark, 1969; Proctor and Cho, 2006), but they cannot necessarily explain away metaphor congruity effects like those we present here. Papers attempting to challenge metaphor theory on the basis of polarity correspondence raise an important concern: “metaphor congruity effects” should not be interpreted as support for metaphor theory, unequivocally, if the data can be explained equally well in terms of another theory, such as polarity correspondence (Lakens, 2012; Lynott and Coventry, 2013). Yet, the conclusions of these papers should be reconsidered: the method Lakens (2012) proposed is not, in fact, capable of disconfirming the predictions of metaphor theory. It is, however, capable of disconfirming the predictions of polarity correspondence, as it did in the present study.

Our findings, which cannot be explained in terms of markedness/polarity correspondence, corroborate previous studies suggesting that people not only talk about musical pitch metaphorically but also think about it metaphorically, activating the particular kind of spatial representation that is encoded in their linguistic metaphors (Rusconi et al., 2006; Evans and Treisman, 2010; Walker et al., 2010; Dolscheid et al., 2013).

## Conclusion

Metaphor-congruity effects have been challenged by a markedness, or polarity-correspondence-based account, claiming that binary response compatibility effects may be better explained by markedness than by metaphorical thinking (Lakens, 2012; Lynott and Coventry, 2013). In binary compatibility experiments, metaphor and polarity are often hard to distinguish. Yet, here we show that when metaphor and polarity are juxtaposed, the congruity effects support metaphorical thinking, not markedness/polarity correspondence.

Furthermore, these results show that it is not necessary to use polysemous words to produce source-target congruity effects (i.e., words that can refer to both the metaphorical source and target domains, such as space and pitch). Words that activate a vertical schema (e.g., tall/short) produce a space-pitch congruity effect despite being lexically inappropriate to describe pitch. Words that activate other spatial schemas (e.g., front/back, big/small) do not produce any space-pitch congruity effect, despite naming the poles of other marked spatial continuums. Together, these results indicate that activating the appropriate spatial schema is the “active ingredient” in space-pitch congruity effects—not polysemy, or markedness/polarity correspondence—supporting theories of metaphorical mental representation.

### Conflict of Interest Statement

The authors declare that the research was conducted in the absence of any commercial or financial relationships that could be construed as a potential conflict of interest.

## Footnotes

1 http://audacity.sourceforge.net/

2 http://www.r-project.org/

3 http://lsa.colorado.edu/
